# Surgery

**Published:** 1899-09

**Authors:** Thomas Carwardine


### SURGERY.

**Catheterisation of the ureters** is of extreme value in certain cases, but requires patience and practice in its performance. It probably has dangers of its own, although Casper thinks that with ordinary care the dangers are very few. He has catheterised the ureters over five hundred times without detecting any infection of the kidney thereby. He insists on the necessity of every precaution being taken, and only employing the method when it is really necessary for diagnosis and treatment.[3] David Newman is of opinion that the fullest possible information should be obtained by the cystoscope before resorting to catheterisation of the ureters as a last resort.[4] All are agreed that it should never be done if the bladder be septic.

Catheterisation of the ureters **in the female** has been rendered possible by Kelly's tubes. In a case of pyuria recently under my care, I was able to draw off turbid urine from one ureter which was found to contain tubercle bacilli. Urine from the other

[3] *Brit. M. J.*, 1898, ii. 1412. [4] *Ibid.*, 1411.

kidney was healthy. This proved a certain and only guide, and the corresponding kidney was found to be tuberculous in an early stage. Briefly the method consists in placing the patient in the genu-pectoral position, or the dorsal position with the pelvis well raised, gauging the urethra, passing a speculum of corresponding calibre, deflecting it 30° to one or other side of the middle line, and searching for the ureteral papilla which betrays its position by an intermittent gush of urine. The orifice can be detected by a probe and the ureteral catheter passed in, often several inches and sometimes up to the pelvis of the kidney.

Catheterisation of the ureters **in the male** is beset with many difficulties. Dr. Willy Meyer has done much work in this direction with instruments based on the principle of Nitze's cystoscope, although he prefers Casper's model[1] of ureter-cystoscope. The essentials are, that the urethral calibre must be adequate, the bladder must have a capacity of at least four ounces, and the fluid in the bladder must be and remain perfectly transparent. The patient is made to imbibe freely beforehand to increase the quantity of urine, then the following processes are gone through: (*a*) The bladder is washed out, cocainised and injected with six ounces of clear fluid. (*b*) The instrument is introduced, afterwards fully closing the lid. (*c*) The ureteral opening is approached, letting it appear at the very end of the cystoscopic picture. (*d*) The catheter is pushed forward parallel with the ureter for one or two inches. (*e*) The wire mandrel is withdrawn, and then the urine collected. Casper's cystoscope permits lateral deviation of the ureteral catheter; but a great improvement seems to be effected in Albarran's instrument, in which the projected bent catheter can be flexed or extended, and so insinuated into the ureteral orifice with greater precision.[2] The application by Howard Kelly of his method to the male sex is strikingly novel, and very welcome in the fact that it does away with optical complications[3] which are always fatiguing. The speculum differs from the female type chiefly in its greater length; viz., sixteen centimetres. The patient assumes the knee-chest position after the instrument has been introduced, the penis occupying an attitude of retroversion between the legs. The left forefinger or a speculum is inserted into the rectum to admit air. When the obturator is withdrawn the bladder also fills with air. The ureteral orifices are then inspected and dealt with as in the female. Kelly has devised a mirror on a handle for inspecting the bladder through the speculum, resembling a small laryngoscope mirror.

**Analysis** of the small quantities of urine obtainable **necessitates special methods.** Dr. Frederic E. Sondern estimates a sample of six cubic centimetres in the following way:—The

1 *Med. Rec.*, 1897, li. 613.

2 *Rev. de Gynéc. et de Chir. abd.*, 1897, i. 457; quoted by Valentine, *Med. Rec.*, 1898, liii. 573.

3 *Ann. Surg.*, 1898, xxvii. 475.

specific gravity is estimated by a Westphal's specific gravity balance, and then the sediment obtained for microscopical and bacteriological examinations by centrifugalisation. One cubic centimetre of the urine is used to test for albumin, and one for the Esbach's test. If albumin be present, the remainder is boiled and filtered. Of the filtrate, one cubic centimetre is used to test for urea, half a cubic centimetre for sugar, and the remainder serves for chlorides and other constituents. Unfortunately the recorded results are discounted by fanciful observation such as "catarrh of the renal pelvis, probably a very moderate pyelitis and the cause of a very moderate renal hyperæmia; moderate chronic cystitis; excessive crystalline deposit, allowing suspicion of renal stone."[1] This method may take as long as two hours to carry out and there appears to be some risk of false passage. One so called "perfectly successful catheterism of both ureters" involved a negative result on one side, and the catheter returned with the tip bent and the eye blocked by a clot.[2]

**Air-inflation of hollow viscera** has been employed in a passive way by Howard Kelly as an essential to his method of cystoscopy, and more recently by others for the active distension of hollow organs:

1. *The Bladder.*—This is passively distended by Kelly's method, both in the male and female, as soon as the obturator is withdrawn. Cystoscopy by straight tubes was employed by Desormeaux in 1865, but the passive air-distension is the special feature of Kelly's method. Active distension of the bladder by air instead of by water has been advocated by Dr. A. T. Bristow[3] and others. Keen first employed the method in 1890. It is claimed that air distension raises the peritoneum from the symphysis at least an inch more than water-distension does. One disadvantage in its employment formerly was the difficulty in estimating the amount of distension. This has been overcome by an ingenious device by Dr. William Jepson.[4] It consists in effect of two graduated bottles, one above the other, with necessary stopcocks. The lower one contains air, and is connected from its upper part by a soft tube to the catheter. The upper bottle contains water, and is connected with the lower bottle by a tube with stopcock. When it is required to distend the bladder with air, water is allowed to flow from the upper to the lower bottle, thus displacing an equal quantity of air into the bladder. The bottles can be raised or lowered to meet hydrostatic requirements. The chief points in this are, that the force is well regulated and perfectly controlled, and the air in the bladder is under a known pressure, but not rigidly confined. This is obviously safer than using a pneumatic bicycle pump, since any extra tension in the bladder expends itself in the column of water above.

[1] Meyer, *Loc. cit.* [2] *N. York M. J.*, 1897, lxv. 324.
[3] *Ann. Surg.*, 1893, xvii. 667. [4] *Ibid.*, 1898, xxviii. 358.

2. *The Rectum.*—Kelly employs passive distensions of the rectum in his method of cystoscopy to depress the base of the bladder. It is particularly important for the male. Active distension was first used by Petersen in 1880 by means of his india-rubber bag, as an adjunct to suprapubic cystotomy. Kelly's tubes are now employed as proctoscopes with passive air-distension of the rectum, and Dr. James Tuttle speaks highly of them.[1] He claims even to have seen twenty to thirty-five inches up the lower gut by their means. By an ingenious method of using the fingers as retractors, combined with passive atmospheric dilatation of the rectum in the knee-chest position, Dr. T. C. Martin states that six or eight inches of the rectum can be easily inspected. The anæsthetised patient is balanced in position by an assistant, while the surgeon closes his fists, crosses his hands, and inserts both index fingers well into the rectum, retracting its walls as far apart as possible.[2]

3. *Gall-bladder.*—Weller Van Hook has recently advocated active air-distension in operations on the gall-bladder and ducts. He fits a nozzle into the gall-bladder, through which he inflates. The air acts as a probe and quickly finds its way into the ducts. It is of advantage in locating the seat of mischief, and subsequently in testing the accuracy of the suturing of the duct.[3]

**Operative treatment of cirrhosis of the liver.** An ingenious and somewhat successful attempt was made in 1896 by Dr. Drummond and Mr. Rutherford Morison, of Newcastle, to re-establish the portal circulation in cirrhosis by obtaining adhesions between the liver, spleen and omentum, and the parietes.[4] This was effected by vigorously rubbing the viscera and parietes with sponges. In one case the operation failed, in another it was very successful. It is premature to indicate the possibilities of this procedure, but of seven published cases two recovered, two were not improved, and three died from shock or sepsis. Weir has gone carefully into the matter, and cites a case, which, however, died on the 5th day from general peritonitis.[5] It appears that Eck, experimenting in 1877, made a series of anastomoses between the portal and systemic circulations in animals: but the results were disastrous, for seven out of the eight animals died within a week of the operation, Moreover, Hahn, repeating the experiments in 1892, found that in animals which survived for a time, the portal blood, passing directly into the systemic circulation, appeared to act as a direct poison, causing excitement, convulsions, coma, and death.

The toilet of the peritoneum, essential in its day, has largely given way before a knowledge of the capacity of the peritoneum

[1] *Med. Rec.*, 1898, liii. 527. [2] *Columbus M. J.*, May 17, 1898.
[3] *Ann. Surg.*, 1899, xxix. 137.
[4] *Brit. M. J.*, 1896, ii. 728. [5] *Med. Rec.*, 1899, lv. 149.

for doing its own scavenger's work. Three cases illustrating the remarkable **tolerance of the peritoneum** have been recently recorded. One was that of a boy of 15, who was gored by a bull, about two inches above Poupart's ligament. He arrived six hours afterwards with 1½ ft. of intestine protruding, smeared over with cow-dung, and covered with various leaves. The parts were cleansed, the peritoneal rents sewn up, the intestines returned and less than three weeks afterwards the lad went home cured.[1] A similar case is that of a woman aged 42 years, who was tossed by a cow early one evening and seen several hours afterwards. Some twenty feet of intestine were protruding from the wound, and when they were returned she expressed herself as better, "now her bowels were in their places again." The wound united by first intention, the temperature never reached 100° and she got up in less than a month.[2] The third case concerned a dissolute married woman of 29. A year previously she had had an abdominal operation preformed, and now the surgeon was sent for as she had "burst" in one of the slums. The surgeon regarded this with such incredulity that he did not visit her till the afternoon of the next day, when he found the woman lying on the floor in the corner of a common living room amongst a heap of straw and filthy rags. She had indeed "burst," and in front of her was a heap of small intestines which had remained amongst the straw and dirt for twenty-four hours. The bowels were washed with ordinary water drawn from a neighbouring tap, the guts were returned and a drainage-tube placed in the lower angle of the wound. She made a rapid and uninterrupted recovery and remained free from recurrence of hernia for years.[3]

The great disadvantage of various bobbins and buttons for **intestinal anastomosis** is that they remain *in situ*, as foreign bodies, for some time after the operation. Dr. Laplace has recently introduced ingenious forceps, by which the advantages of a bobbin or button are secured as regards facility of suture, and those of simple suture by their immediate removal at the end of the operation.[4] The forceps combine to make two handled rings which are introduced into the intestine to be anastomosed, for fixation and support. Each part is separable into two halves so as to be extricated just before completion of suturing. A new feature thus introduced is the control of the operation given by means of the handles. The forceps are duplex, consisting of a semicircle on each half but when locked together they open as two rings, very much like a pair of tongue forceps. Thus, after completion of suturing, the clamp on the forceps is removed, four sickle-shaped pieces have to be taken out and the site they occupied sutured.

[1] *Brit. M. J.*, 1898, ii. 897. [2] *Lancet*, 1899, i. 290.
[3] *Ibid.*, 1898, ii. 74. [4] *Ann. Surg.*, 1899, xxix. 297.

One ring of the forceps is inserted into the gut above, another into the gut below, and they are then approximated. The suturing is carried out with ease, and before the final stitch is tied the portions of the forceps are removed. The instrument is made in five sizes, the smallest being suitable for gall-bladder surgery. By its means end-to-end or lateral anastomosis, and also invagination, may be rapidly performed.

THOMAS CARWARDINE.

